# Oral health of adult patients undergoing hematopoietic cell transplantation. Pre-transplant assessment and care

**DOI:** 10.1007/s00277-017-2932-y

**Published:** 2017-02-13

**Authors:** Agnieszka Bogusławska-Kapała, Kazimierz Hałaburda, Ewa Rusyan, Hubert Gołąbek, Izabela Strużycka

**Affiliations:** 1 Department of Comprehensive Dental Care, Medical University of Warsaw, Miodowa 18, 00-246 Warszawa, Poland; 2 Department of Hematopoietic Stem Cell Transplantation, Institute of Haematology and Transfusion Medicine, Indiry Gandhi 14, 02-776 Warszawa, Poland; 3 Department of Conservative Dentistry, Medical University of Warsaw, Miodowa 18, 00-246 Warszawa, Poland

**Keywords:** Hematopietic cell transplantation, Pre-transplant period, Oral health, Dental proceedings

## Abstract

Hematopoietic cell transplantation (HCT) is now one of the frequent procedures used for treatment of malignant and non-malignant blood diseases, autoimmune disorders, and certain solid tumors. Despite improvements of therapeutic protocols, HCT still carries a high risk of non-relapse mortality due to early and late complications. Side effects of the therapy regimen frequently occur in the oral cavity and often significantly decrease the patients’ quality of life. The complications may result from or may be exacerbated by improper oral preparation of the patient before transplantation. Therefore, it is mandatory that all patients referred to HCT undergo thorough dental examination and receive appropriate treatment before the procedure. It is also very important to develop an individual post-transplantation oral care protocol with special concerns to oral hygiene before implementation of the conditioning. This paper presents a review of dental management methods intended for patients before HCT proposed in literature as well as recommendations based on the experience of the Department of Comprehensive Dental Care and the Department of Conservative Dentistry of Warsaw Medical University and the Warsaw Institute of Hematology and Blood Transfusion. The article pays special attention to the problem of potential foci of infection and bleeding. It also presents protocol of oral hygiene in post-transplantation period, used by patients of Warsaw Medical University and Institute of Hematology and Blood Transfusion.

## Introduction

Hematopoietic cell transplantation (HCT) is now one of the frequent procedures used for the treatment of malignant and non-malignant blood diseases, autoimmune disorders, and certain solid tumors [1]. According to the report of the European Society for Blood and Marrow Transplantation, there were 34,809 first-time transplants reported to the registry in 2013. The number included 19,859 autologous transplants (57%) and 14,950 (43%) allogeneic ones. Myeloablative conditioning with or without TBI was used in 60% of the total allogeneic HCT (allo-HCT) [2].

Despite improvements of therapeutic protocols, HCT still carries a high risk of non-relapse mortality due to early and late complications. Among those, infections play an important role, especially in early post-transplantation period. These and other complications are mostly related to allogeneic HCT, which requires administration of immunosuppressive drugs for several months and in some patients permanently. In addition to neutropenia and immunosuppressive treatment, specific complications like graft versus host disease (GvHD) contribute to profound and long-lasting impairment of immunity. Conditioning for autologus transplantation (auto-HCT) induces only transient neutropenia and relatively mild alteration of host defense. Severe mucosal injury after auto-HCT usually resolves without long-term sequelae. Compared to allo-HCT, in auto-HCT, the risk of delayed or persistent serious complications within the oral cavity, including infections, is substantially lower [1].

In the oral cavity acute, delayed and late side effects of the transplantation regimen affect approximately 80% of the patients [3–5]. In the early post-transplantation period, mucositis resulting from conditioning is the main clinical symptom which varies in degree and severity and depends primarily on the intensity of conditioning regimen.

Damage of the oral mucosa together with profound myelo- and immunosuppression after transplantation may lead to local and systemic infections. Other side effects in the early period may include bleeding due to thrombocytopenia as well as pain, nutrition, and articulation impairment caused by mucositis. In the first year after transplantation, oral cavity is affected by delayed complications like hyposalivation, taste disorders, and dentin hypersensitivity, which often importantly decrease the patients’ quality of life. Additionally, there still remains a high risk of infection and bleeding in this period. At later stages, long-term complications, from which oral chronic GvHD is the most important, may contribute to sicca syndrome, mastication impairment, malabsorption syndrome, and *lichen planus* lesions in some cases. Also, other complications like advanced periodontal disease, rapid caries, and taste disorders are common. It is significant to remember about the risk of secondary cancers [6]. Although oral problems can compromise the treatment protocol and in some cases affect the chance of survival, they are still underestimated and undertreated [5, 6].

According to literature in line with clinical experience and the authors’ own practice, oral complications may result from or may be exacerbated by improper dental preparation of the patient before transplantation and inadequate post-transplant oral care [7–9]. Therefore, all patients waiting for transplantation, definitely have to undergo thorough dental examination and receive appropriate treatment in adequate time before the procedure.

This paper presents a review of methods for dental management of patients before HCT proposed in literature as well as recommendations based on the experience of authors of the publication.

## General consideration regarding oral care protocol before HCT

Detailed medical check-up before transplantation must include a thorough sanitation of oral cavity conducted by an experienced dentist who should preferably be a permanent member of the transplantation team [10, 11]. It is recommended that dental proceedings should include patient’s examination, establishment of treatment plan, execution of necessary dental treatment, informing patient about possible side effects of the therapy and delivery of oral hygiene instruction, implementation of fluoride prophylaxis, and recommendation of antibacterial oral rinses [6, 12].

## Examination of the patient

The physical examination is carried out as normal. An orthopanthomogram (OPG) of the maxilla and the mandible is indispensible at the time of the first visit. Additional intra-oral x-rays may be ordered if necessary [13, 14]. The dentist should send a written dental report to the treating physician. The optimal time for necessary dental procedures should be specified in this paper.

## Establishment of treatment plan and its limitations

Routine oral cavity pre-transplantation procedures should basically involve identification and elimination of active and potential foci of infection within the stomatognathic system and eradication of local factors that may provoke trauma, pain, and bleeding (Table 1) [3, 6]. Ideally, all existing and potential sources of bleeding and/or pain should be eliminated before the initiation of the conditioning. However, as indicated by our experience, complete sanitation of oral cavity prior to transplantation is not always achievable. The major elements that modify oral treatment plan are the urgency of transplantation, patient’s general health and remission status, peripheral blood counts, nature and complexity of oral disease, patient’s financial resources, motivation, and difficult access to dental services [24].

**Table 1:** Potential foci of infection and local trauma/bleeding factors within the stomatognathic system in patients undergoing HCT [12, 15–23]

Focus of infection	Advanced caries	-Cavities in which excavation may lead to pulpal exposure, -Cavities that can harbor fungal organisms, -Unrestorable carious teeth, -Compromised, large restorations
	Moderate/advanced gingivitis and periodontitis	- Furcation involvement greater than II grade, -Advanced tooth mobility, -Periodontal pockets ≥6 mm, -Advanced recession (loss of two-third bone support)
	Dental-pulp/periapical pathology	-Nonvital/inflamed dental pulp, -Chronic/acute periapical pathology
	Others	-Pericoronitis, -Sub, supra-gingival calculus, -Partially erupted teeth, -Root tips (not fully covered by bone or showing radiolucency), -Cysts, -Mucosal lesions, -Leaking crowns and fillings
Local bleeding factors		-Fractured teeth and/or restorations, -Sharp edges of teeth and/or restorations, -Ill-fitting dentures, -Orthodontic appliances, -Teeth contacting directly to the opposing mucosa

There is a lack of clearly defined oral treatment protocols in the literature regarding indications to selected dental procedures before HCT. This particularly concerns the treatment of teeth with chronic pulpal/periodontal pathology which can be a potential source of infection. The most controversial question is: extraction or treatment? Some authors suggest radical approach. They propose the removal of all teeth with uncertain prognosis as the only choice [6, 25]. Other studies, by contrast, recommend minimally invasive pre-HCT dental treatment, which means that in time constraints, acute pathologies and non restorable teeth should be removed primarily [15, 26–28]. It is emphasized that untreated chronic dental pathologies do not have statistically important influence on post-HCT infections. Tooth extraction is associated with an increased risk of bleeding and also can attract prolonged wound healing, a potential entry for infection [29]. Toljanic et al. [26] conducted a prospective study on patients undergoing chemotherapy and found that the conversion of chronic dental disease to an acute state during chemotherapy does not occur frequently. Yamagata et al. [15] similarly observed that chronic dental pathologies (including periapical radiolucencies less than 5 mm) that were left untreated before HCT do not significantly influence on the health status of HCT patients. This is in agreement with the results found by Melkos et al. [27], Peters et al. [28], and Schuurhuis et al. [30], who stated that untreated chronic periapical foci did not affect the incidence of infection during HCT.

In Warsaw Institute of Hematology and Blood Transfusion, we recommend pre-HCT oral treatment plan primarily determined by the time left for the procedure, general health status of the patients, and toxicity of the adopted conditioning regimen. On this basis, we qualify the specific oral need (e.g., caries, periodontitis, poor oral hygiene) as mandatory or optional. In order to achieve the best results, evaluation should be performed even before the recipient’s final medical check-up. If started early enough, it may help to avoid unnecessary extractions and influence the patient’s quality of life after transplantation. It is preferable to complete all dental treatments at least 2 weeks before the initiation of the conditioning [1, 6]. Unfortunately, the referral for HCT is often urgent due to the compromised medical status of the patient. Also, the initial dental examination often takes place very close to the beginning of HCT procedure. In consequence, time for oral treatment is limited [24]. If there is not enough time to complete sanitation in a clinic, we recommend to concentrate primarily on acute needs: eradication of active infections (e.g., acute periodontal/periapical pathologies) and elimination of potential sources of bleeding and/or pain. Also, non-restorable teeth (i.e., exposed root tips) should be extracted (Table 3, Appendix 1, 2). Elective treatments can be addressed at the time when the patient is in good condition. The toxicity of conditioning regimen is also taken into consideration. The lower risk of general and oral complications allows to reduce indications for teeth extractions and to apply conservative treatment instead. Extended indications for extractions are suggested particularly in case of (1) high risk of early complications, as a result of high-dose cytotoxic conditioning and expected aGvHD; (2) high risk of long-term complications, especially in high risk of cGVHD; and (3) the patient’s poor motivation for oral hygiene (Appendix 3). But, it has to be mentioned that multiple extractions increase the risk of bleeding, infection, delayed wound healing, and possible postponing of medical treatment [16, 27, 29]. We observe that they can also badly affect the long-term health conditions and quality of life, because removal of many teeth may compromise nutrition and have negative consequences for occlusion and esthetic aspects as well.

## Execution of dental procedures

The risk of bacteremia and sepsis is correlated primarily with low neutrocyte count (ANC), but hematology patients even with normal ANC values should be considered for antibiotic prophylaxis. Therefore, the treating physician should determine the need for such prophylaxis at the time of referral for dental treatment [17, 35]. Unless the hematologist proposes differently, recommendations of the American Heart Association should be followed (Table 2) [31].

**Table 2:** Antibiotic prophylaxis before dental procedures (according to the American Heart Association 2007) [31]

60 min before the procedure		
Orally	Amoxicillin	2 g
Allergy to penicillin	Clindamycin Cephalosporin Macrolides	600 mg 2 g 500 mg
Oral administration impossible	Ampicillin Cephalosporin	2 g im/iv 1 g im/iv
Oral administration impossible and allergy to penicillin	Cephalosporin Clindamycin	1 g im/iv 600 mg im/iv

Prophylactic platelet (PLT) transfusions are recommended at PLT concentrations ≤30–40 × 10^9^/L [13, 36]. In thrombocytopenic patients, local hemostatic measures should be implemented apart from PLT transfusions. Intra-alveolar dressings should be avoided (a potential medium for bacterial growth), but sutures are recommended [35, 37]. It should be remembered that non-steroidal anti-inflammatory drugs (NSAIDs) commonly used in dentistry may increase risk of bleeding. In the case of thrombocytopenia, medicaments without anti-aggregation effect should be used (i.e., paracetamol, partial mixed opiate receptor agonists, and mild opioids, such as oxycodone, hydroxycodone codeine, and tramadol) [38]. The proposed procedures are presented in Appendix 1 and 2 and Table 3.

**Table 3:** Recommendations of the minimally invasive dental treatment in a patient scheduled for HCT, based on the literature and the experience of the authors [3, 13, 22, 24, 31–34]

Periodontal diseases	-Removing of calculus and smoothing sharp edges of restorations, prosthetic restorations, and teeth, -Extractions: teeth with periodontal pockets of 6 mm or deeper, teeth affected by endoperiodontic complex with grades II and III furcations, markedly mobile (grade III mobility), -Removing implants with pockets deeper than 6–7 mm
Impacted teeth	-Removing teeth fully or partly erupted but only with signs of acute or chronic inflammation
Orthodontic treatment	-Consider removing braces, Orthodontic treatment can be continued within a year of HCT, if the patient is not on immunosuppression
Prosthetic treatment	-Removing poor, permanent prosthetic replacements Removable prosthetic restorations may continue to be worn depending on the mucous membrane condition (mucositis, xerostomia) and the condition of the denture
Other procedures and recommendations	-Reshaping or removing the teeth contacting opposing mucosa -Treatment of parafunctions

## Oral hygiene instructions

Before initiation of conditioning, patients must be aware of adverse effects of therapy which affect oral cavity. All of them should be explained. Patients have to be instructed about methods of preventing such complications and treatment. Oral hygiene practices must always be adapted to individual needs of the patients and clinical situation. The instruction is delivered both to the patients and their caregivers (family, medical staff). In our clinic, the recommendation are provided both orally and in writing and include information on teeth brushing (type of toothpaste, toothbrushes, accessories such as floss, irrigators), additional methods of topical fluoridation, oral mucosa and lip care (including oral rinses), and the maintenance of prosthetic restorations. Oral hygiene instructions given to patients before the beginning of the transplantation in Warsaw Institute of Hematology and Blood Transfusion are contained in Table 4.

**Table 4:** Oral hygiene instructions intended for patients in Warsaw Institute of Hematology and Blood Transfusion and in the Department of Dentistry of Warsaw Medical University

	Before transplantation	In the hospital	After the hospital
Teeth	*Cleaning teeth*: at least twice a day (after every meal if possible). *Toothbrush*: soft or medium, manual or electric, replaced once a month. *Toothpaste:* without lauryl sulfate. If you do not have problems with gum bleeding, use dental floss, dental sticks, and irrigators.	*Significant thrombocytopenia or if tooth brushing is painful:* Clean with a sterile gauze and gentle antibacterial solution without alcohol (as prescribed). Do not use dental floss. *Without significant thrombocytopenia:* clean at least two times a day (after every meal and before night is indicated). Use the super soft toothbrush, replace once a week and a non-mint toothpaste without lauryl sulfate. Initially, rinse out with saline solution. After the healing of mucosal changes, it is allowed to rinse with running water. Use dental floss once a day. Additional methods of dental care—as prescribed. *If there is the necessity of high-calorie food intake, you have to pay special attention to systematic oral care. It is recommended to avoid sugars and use lactose-free ready-to-feed nutritional products*.	*Cleaning teeth:* at least twice a day (after every meal and before night is recommended). *Toothbrush:* soft or medium, manual or electric, replace once a month. *Toothpaste:* without lauryl sulfate or toothpaste prescribed by the dentist. If you suffer from dental hypersensitivity, use the toothpaste dedicated to this problem. Rinse out with running water. *Additional accessories:* dental floss with fluoride, dental sticks, interdental brushes, and irrigators. *Additional home methods of caries protection (necessary):* -Fluoride rinses without alcohol (at least weekly). Keep it in mouth for 2–3 min, then spit. or -Fluoride gel (twice daily), Do not rinse. Do not eat and drink for an hour after fluoride application. If in doubt, ask the dentist for advice. *During the first year after transplantation, it is recommend to visit your dentist every 3 months or more often if necessary*.
Prosthesis	Show the dentist your dental prosthesis. Sometimes it will have to be replaced with the new one.	Do not use the prosthesis in case of mucositis or use it only during meals. Remove immediately after every meal and clean it with toothbrush and yellow soap. Soak for 1 hour in the antibacterial solution recommended by your dentist. Then wash the prosthesis carefully with the saline solution. Keep it dry.	Use your prosthesis only to meals during the first 3 months after transplantation—especially when mucosal changes exist. Clean the prosthesis after every meal with soft toothbrush and yellow soap. Soak for 1 hour in the antibacterial solution recommended by your dentist. Then, wash the prosthesis carefully with running water. Keep it dry. Remove your prosthesis overnight. Remove the prosthesis before cleaning your own teeth or rinsing the mouth. In case of dryness, you can moisture the inner surface of the prosthesis with an artificial saliva.
Oral mucosa	*Rinsing the mouth:* twice a day with chlorhexidine solution: 0.2% (10 ml) or 0.12% (18 ml). You can dilute the liquid with boiled water (1:1). Start rinsing at least 7 day before the beginning of chemo/radiotherapy. Keep 1 hour distance between irrigation with the chlorhexidine solution and: -Washing your teeth, -Using antifungal agents (especially nystatin), -Eating, drinking.	*Rinsing the mouth:* -Saline solution (every 3 h) or -Baking soda solution (1 teaspoon diluted in 250-ml slightly warm boiled water, every 3 h). In case of severe dryness, use additional rinses (as prescribed, e.g., artificial saliva). Avoid mouthwashes containing alcohol, citric acid, and glycerol. *Cleaning the tongue:* soft toothbrush or gauze with saline or baking soda solutions. Your doctor can order rinsing your mouth with antifungal agents. Remember: -To remove your prosthesis before rinsing, -Not to eat, drink, wash your teeth, or use other medical remedies for at least an hour after the application. You can lubricate the inner surface of the prosthesis with the antifungal medicament. *Other recommendations:* -Chew your food gently, -Rinse your mouth after vomiting, -Avoid abnormal behaviors (e.g., biting the oral mucosa with the teeth), -Avoid coffee, tobacco, sweetened drinks (cola, juices, tea), and sweet, sticky foods, dried spices.	In case of oral dryness: *Stimulating the saliva secretion:* -Chewing gum without sugar (for example with xylitol), -Candies without sugar, -A piece of frozen fresh pineapple or canned pineapple but rinsed carefully with water first. Do not use the pineapple in case of mucositis. *Moisturizing the mouth:* -Frequent sipping of still water, keeping in the mouth for a while. You can add few drops of fresh lime juice, -Moisturizing agents (oral balance, caphosol, artificial saliva), 4–10 times a day, -Rinsing with infusion of linseed, -Air humidification at home.
Lips	Use vaseline, allantoin, or vitamin A ointments.	Clean with the gauze with gentle disinfectants (e.g., baking soda). Take special care of your mouth corners! Use vaseline, allantoin, or vitamin A ointments.	Use vaseline, allantoin, or vitamin A ointments.

## Implementation of fluoride prophylaxis

Patients undergoing HCT, and especially allo-HCT, have a high risk of developing caries. This is primarily due to inadequate secretion of saliva, which affects more than 50% of patients in the post-transplant period [18]. Also, mucositis and general clinical condition of the patient often preclude thorough teeth and oral cavity cleaning. Therefore, in protocol developed in our institution, we recommend introducing additional methods of dental hard tissues remineralization well before transplantation procedure. Fluoride prophylaxis follows the recommendations of the American Dental Association (ADA) dedicated for patients 6 years or older being at an elevated risk of dental caries. The protocol includes professionally applied topical fluoride agents: (1) 2.26% fluoride varnish (at least every 3–6 months) or (2) 1.23% fluoride (APF) gel for 4 min (at least every 3–6 months) and prescription-strength, home-use measures: (1) a prescription-strength, home-use 0.5% fluoride gel or paste (twice daily) or (2) 0.09% fluoride mouthrinse (at least weekly) [39]. Non-mint-flavored toothpaste with fluoride but free from sodium lauryl sulfate, which may irritate oral soft tissues, is recommended. Fluoride varnishes should be avoided in case of irritation of the mucosa [6, 40].

## Recommendation of antibacterial oral rinses

Because of an increased risk of oral infections, introduction of local antimicrobial solutions is suggested. Our patients start using mouthwash measures 7–14 days prior to the conditioning. Alcohol-free rinses with 0.12–0.2% chlorhexidine digluconate solution are advisable. The patients should remember to make at least an hour break between the application of antibacterial rinses and tooth brushing or use of locally acting antifungal suspensions (nystatin, amphotericin) [6].

## Discussion

It is a widely recognized fact that general oral health of most patients undergoing HCT is not satisfactory and reflects the status in the societies they come from. Although some of the acute oral disorders resulting from the therapy may be reduced, in many cases, they remain unavoidable. Also, the significant impact of long-term complications may negatively affect patient’s quality of life. Therefore, this unique group of patients requires increased awareness and recognition to promote the appropriate oral prophylaxis and care. Unfortunately, not all patients undergo specialist dental examination at the pre-HCT stage [19, 41]. According to Barker [32], only 40% of the patients receive this type of examination. Also, there are few algorithms of dental management based on reliable clinical studies targeted at adult patients undergoing transplantation [3, 5, 6, 42]. Available protocols based on standards of individual transplant centers are sometimes contradictory and imprecise. Various authors differ in their opinions especially when it comes to defining potential foci of infection within the stomatognathic system, and whether it is mandatory for them to be eliminated before conditioning chemo/radiotherapy is commenced. Some of them are in favor of radical oral sanitation and recommend removal of all existing and potential sources of infection [8, 16, 43–46]. Eradicating all active and potential foci of infection may significantly lower the risk of local and systemic complications associated with immunosuppression (from 40 to 12%) [16]. Other authors however are in favor of limited, minimally invasive dental treatment before HCT [15, 24]. They refer to disadvantageous aspects of radical approach, as it may delay treatment of the underlying disease. It is emphasized that invasive procedures in itself carry a risk of infection and increases the risk of prolonged bleeding [15, 47]. Moreover, radical sanitation often means extraction of teeth that otherwise would be conservatively treated, thus, harming the patient [28]. Missing teeth may contribute to alimentary disorders, since wearing prosthetic restorations following transplant is often problematic.

Before initiation of conditioning regimen, the patient must be made aware of adverse effects of the therapy which affect oral cavity; they also have to be instructed in methods of their prevention and treatment. According to reports in literature, only 30% of the recipients are given this kind of information prior to HCT [48]. Detailed oral hygiene instructions delivered to the patients and their caregivers are an important part of dental pre-HCT treatment. The patients should be motivated to begin appropriate hygiene regimen several weeks before transplantation, as it may alleviate potential oral cavity complications during and after transplantation. At pre-transplant stage, the patients should also be advised of the necessity of frequent dental check-ups, especially in the first year after the transplant procedure.

## Conclusion

Patients undergoing hematopoietic stem cell transplantation often suffer from oral ailments that are a consequence of the basic illness or therapy. These complications may compromise health or even be life-threatening during HCT. Adequate dental treatment may decrease this risk. So far, there are no commonly accepted protocols of dental examination and treatment of patients before HCT. Each transplant center should design and implement a dental care protocol based on available literature and own clinical experience. For best results, experienced dental practitioners must be permanently included in transplantation teams.

## Appendix 1

Possible protocol for minimally invasive treatment of caries/non carious lesions in adult patients prior to HCT, based on the literature and the experience of the authors (considering the severity of lesion, time for completing the dental treatment, and the medical status of the patient) [3, 15, 22, 26, 36, 46, 47, 49, 50].

1. Criteria for caries progression [3, 26, 51]**:**
  - Initial caries lesion: limited to the enamel or cementum or very outermost layer of dentin on the root surface
  - Moderate caries lesion: visible signs of enamel breakdown, the dentin is moderately demineralised, shallow cavitation
  - Advanced caries lesion: dentin is exposed, deep cavitation: without symptoms of irreversible pulpitis (clinical/radiographic examination) or with symptoms of irreversible pulpitis (clinical/radiographic examination)
2. Non-carious lesion: the lesion of dental hard tissues of non cariogenic origin (attritions, erosions, abfractions, abrasions)
3. The assessment of time for dental treatment before the conditioning regimen:
  - Sufficient time for completing the procedure: the final dental procedure can be successfully terminated (e.g., root canal therapy with permanent obturation). The risk of local/general complications of the treatment is taken into consideration. No endodontic treatment unless patient has 7 days from the completion of endodontic therapy to onset of myelosuppression (<1000–2000 granulocytes/mm3, 3, 27]
  - Insufficient time left for competing the procedure: the final dental procedure cannot be successfully terminated, or there are acute needs to be cured first. The risk of local/general complications of the treatment is taken into consideration
4. Risk of dental treatment, concerning general health status of the pre-HCT patient:
  - High risk patient: neutropenic and/or thrombocytopenic patient,
  - Moderate risk patient: myelosuppression/ immunosuppression due to chemotherapy,
  - Low risk patient: no evidence of malignancy and myelosuppression [9, 20]
5. ART (atraumatic restorative treatment): the approach that involves the use of hand instruments only to remove carious tooth substance and then restoring the cavity and sealing any adjacent enamel fissures with a glass ionomer restorative cement (GIC) [49]

## Appendix 2

The protocol of minimally invasive treatment proposed for pulpal/periapical lesions in patients prior to HCT considering the severity of pulpal pathology, time for completing endodontic treatment, and the medical status of the patient [3, 22, 26, 35, 36, 46, 47]

1. The assessment of time for dental treatment before the conditioning regimen:
  Sufficient time for completing the procedure: the final dental procedure can be successfully terminated (e.g., root canal therapy with permanent obturation). The risk of local/general complications of the treatment is taken into consideration. No endodontic treatment unless patient has 7 days from the completion of endodontic therapy to onset of myelosuppression (<1000–2000 granulocytes/mm3, 3, 27]
  Insufficient time left for competing the procedure: the final dental procedure cannot be successfully terminated, or there are acute needs to be cured first. The risk of local/general complications of the treatment is taken into consideration.
2. Risk of dental treatment, concerning general health status of the pre-HCT patient:
  High risk patient: neutropenic and/or thrombocytopenic patient,
  Moderate risk patient: myelosuppression/immunosuppression due to chemotherapy,
  Low risk patient: no evidence of malignancy and myelosuppression [9, 20]

## Appendix 3

The protocol of minimally invasive treatment for pulpal/periapical lesions in patients prior to HCT considering the risk of oral and general complications as a result of the toxicity of the conditioning proposed by Warsaw Institute of Hematology and Blood Transfusion.

1. Moderate risk of complications in case of reduced intensity conditioning
2. High risk of complications in case of high-dose cytotoxic conditioning, high risk of long-term complications, especially in high risk of GVHD.
